# High-throughput in situ single particle X-ray imaging of dehydrating viral capsids

**DOI:** 10.1038/s41377-026-02262-0

**Published:** 2026-06-23

**Authors:** Abhishek Mall, Anna Munke, Parichita Mazumder, Zhou Shen, Johan Bielecki, Juncheng E, Armando D. Estillore, Chan Kim, Romain Letrun, Jannik Lübke, Safi Rafie-Zinedine, Adam Round, Ekaterina Round, Michael Rütten, Amit K. Samanta, Abhisakh Sarma, Tokushi Sato, Florian Schulz, Carolin Seuring, Tamme Wollweber, Lena Worbs, Patrik Vagovic, Richard Bean, Adrian P. Mancuso, Ne-Te Duane Loh, Tobias Beck, Jochen Küpper, Filipe R.N.C. Maia, Henry N. Chapman, Kartik Ayyer

**Affiliations:** 1 Max Planck Institute for the Structure and Dynamics of Matter, 22761 Hamburg, Germany; 2 Center for Free Electron Laser Science, Deutsches Elektronen Synchrotron (DESY), 22607 Hamburg, Germany; 3 Laboratory of Molecular Biophysics, Department of Cell and Molecular Biology, Uppsala University, Uppsala, SE-75124 Sweden; 4 European XFEL, Holzkoppel 4, 22869 Schenefeld, Germany; 5 Institute of Biomaterials and Biomolecular Systems, University of Stuttgart, Pfaffenwaldring 57, 70569 Stuttgart, Germany; 6 Institute of Physical Chemistry, Universität Hamburg, Grindelallee 117, 20146 Hamburg, Germany; 7 The Hamburg Center for Ultrafast Imaging, Universität Hamburg, 22761 Hamburg, Germany; 8 Centre for Structural Systems Biology (CSSB), Notkestraße 85, 22607 Hamburg, Germany; 9 Department of Physics, Universität Hamburg, Luruper Chaussee 149, 22761 Hamburg, Germany; 10 Diamond Light Source, Harwell Science and Innovation Campus, Didcot, Oxfordshire OX11 0DE UK; 11 Department of Chemistry and Physics, La Trobe Institute for Molecular Science, La Trobe University, Melbourne, VIC 3086 Australia; 12 National University of Singapore (NUS), Department of Physics/Faculty of Science, 2 Science Drive 3, Singapore, 117542 Singapore; 13 Department of Chemistry, Universität Hamburg, 20146 Hamburg, Germany; 14 NERSC, Lawrence Berkeley National Laboratory, Berkeley, CA 94720 USA

**Keywords:** Imaging and sensing, X-rays, Microscopy

## Abstract

Single-stranded RNA viruses co-assemble their capsid with the genome, and variations in capsid structures can have significant functional relevance. In particular, viruses need to respond to a dehydrating environment to prevent genomic degradation and remain active upon rehydration. Theoretical work has predicted low-energy buckling transitions in icosahedral capsids, which could protect the virus from further dehydration. However, there has been no direct experimental evidence, nor a molecular mechanism, for such behavior. Here, we observe this transition using X-ray single particle imaging of MS2 bacteriophages after aerosolization. Using a combination of machine learning tools, we classify hundreds of thousands of single-particle diffraction patterns to learn the structural landscape of the capsid morphology as a function of time spent in the aerosol phase. We found a previously unreported compact conformation as well as intermediate structures that suggest an incoherent buckling transition that does not preserve icosahedral symmetry. Finally, we propose a mechanism for this buckling, where a single 19-residue loop is destabilized, leading to the large observed morphological change. Our results provide experimental evidence for a mechanism by which viral capsids may protect themselves from dehydration upon aerosolization. In the process, these findings also demonstrate the power of single-particle X-ray imaging and machine learning methods in studying biomolecular structural dynamics.

## Introduction

Viral capsids assemble optimally to prioritize the protection and efficient packaging of the genome^1,2^. This ensures the survival of the virus and facilitates interactions with a host to maintain infectivity. Most spherical viruses in nature assemble their capsids with icosahedral symmetry, characterized by a triangulation number (*T*): the number of structural subunits forming the triangular facets of the icosahedron^3^. For instance, the MS2 bacteriophage, a 27 nm single-stranded RNA virus infecting *Escherichia coli* bacteria (*E. coli*), is a non-enveloped virus with a *T* = 3 icosahedral capsid structure^4^. With non-genomic RNA, the capsid protein can also assemble into *T* = 4 as well as hybrid capsids between these two triangulation numbers^5^. Furthermore, covalent dimerization of the coat protein in MS2 can lead to an octahedral structure under certain buffer conditions^6^.

The variability in capsid structures and symmetry-breaking in icosahedral capsids can potentially affect infectivity, and has been well-studied in the context of viral maturation^2^. The shape of the capsids is determined by elastic properties such as stretching and bending energies, spontaneous curvature, and chirality. The transition from smooth to faceted shapes in icosahedral capsid shells corresponds to a soft-mode buckling transition, driven by bending stiffness^7^. Continuum elasticity theory attributes shape transitions in capsids with non-icosahedral symmetries to a trade-off between stretching and bending energies^8^. Moreover, the elastic responses to external forces elucidate the mechanical stability and rupture behavior of both empty and filled viral capsids^9^.

Understanding the intricate and non-trivial variations in viral capsid structure is essential for unraveling the fundamental processes driving viral infectivity and hardiness. In this study, we approach this problem using the emerging technique of single-particle imaging (SPI) at an X-ray free-electron laser (XFEL) source. This is a powerful method for probing the structures of nanoscale systems^10–12^. In these experiments, extremely bright, ultrashort, and coherent X-ray pulses from XFELs interact with copies of isolated single particles in random orientations one at a time. This process generates millions of diffraction patterns, each from a single viral particle. Machine learning approaches, including unsupervised methods^13,14^, are employed to identify diffraction patterns scattered from the target object amid contaminants, aggregates, and outliers. This is followed by orientation determination and phase retrieval to obtain the electron density of the average particle^15–17^. Since each measurement is made on an individual particle, one additionally has the opportunity to classify them and obtain not only the average structure, but also the landscape of structural variations^11–13,18^.

The short pulses of an XFEL also enable time-resolved SPI experiments to investigate ultrafast phenomena and structural dynamics in ensembles of particles at the nanoscale. This progression has enabled the exploration of ultrafast photo-induced dynamics^19^, resolving the non-equilibrium shape distributions^12^, retrieving the 3D morphology of polyhedral particles^20^, melting to explosive disintegration of nanoparticles^21^, demonstrating diffraction before destruction at the protein scale^22^, and retrieving structures of heterogeneous nanoparticles^11^.

In this work, we explore and analyze the structural dynamics of MS2 bacteriophage viruses after aerosolisation. In the process of being transported to the XFEL beam, the aerosol droplets are continuously drying, simulating the natural dehydration process^23–25^. Dehydrated viruses have been the target of studies since the earliest days of macromolecular structural studies^26^. These studies used electron microscopy^27^ and crystallography^28^ to study dried virus crystals, reporting a reduction in the diameter compared to the hydrated case, but still with a symmetric structure. This still left the question open about the structure of the intermediate stages during dehydration, and the possibility that asymmetries and heterogeneity introduced by the drying process were averaged over. Another approach applied to study dehydrated viruses is using atomic force microscopy (AFM)^29,30^. These studies showed mostly icosahedral structures and, in certain cases, structures resembling those that have been produced in wet conditions under high salt concentrations.

In the current experiment, the aerosolized particles are probed using the XFEL at random degrees of dehydration to produce single-particle diffraction patterns. Using a combination of maximum likelihood and deep learning techniques, we map the collected diffraction data from the ensemble of MS2 capsids to a continuous structural landscape. One can then observe viral capsid structures ranging from the fully-hydrated state to a previously unobserved capsid form with full coverage of intermediate structures. This data then enabled us to hypothesize a molecular mechanism for the observed conformational changes, which seems to protect the genome from further dehydration. In the process, we also show how the combination of machine learning methods with high-throughput SPI measurements at XFELs can be used to understand the conformational landscape and dynamics of biomolecules in a fairly general manner.

## Results

**Fig. 1:**
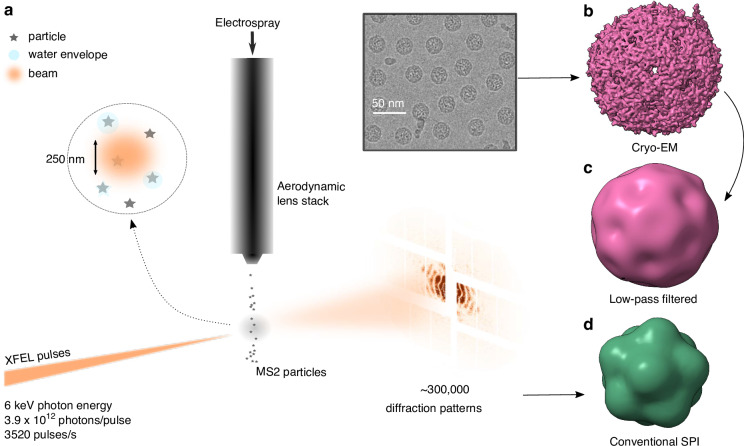
X-ray SPI experiment. **a** MS2 bacteriophage particles, ∼27 nm in diameter, were aerosolized using an electrospray and focused with an aerodynamic lens stack to the interaction region within the X-ray beam of 250 × 250 nm^2^ focus. The top inset shows a representative cryo-electron microscopy (cryo-EM) micrograph of the particles. **b** The 3D structure of MS2 capsid, determined by cryo-EM (resolution 0.49 nm), served as the control for the subsequent X-ray SPI experiment. **c** The same cryo-EM structure was low-pass filtered to the resolution of the conventional X-ray SPI structure. **d** The structure retrieved from diffraction data (6.1 nm resolution) using the conventional analysis pipeline is notably different from the cryo-EM structure in (**c**)

MS2 bacteriophage particles in an aqueous buffer (sample preparation details in the “Methods” section) were aerosolised and sequentially injected into the X-ray beam interaction region using an electrospray-ionization aerodynamic-lens-stack sample delivery system^31^, as shown in Fig. 1a. Diffraction patterns were collected at an average rate of 3520 frames/s for an integrated collection time of 3.6 h with a hit ratio of around 0.7%. Frames with diffraction from particles were detected by setting a threshold on the scattered signal. A total of 287,168 potential hit diffraction patterns were identified, containing 4873 photons per pattern on average, in the resolution range of 48–3 nm. The average non-hit frame contained 1014 photons in the same range.

The highly noise-tolerant EMC algorithm^32^ can be used to categorize and orient diffraction frames with only a few photons^16,33,34^. We employed the *Dragonfly* software^15^, to perform two-dimensional (2D) classification using this algorithm. This procedure generated multiple 2D intensity models of diffraction patterns in the detector plane^11^ by determining the in-plane rotation angle and relative incident fluence of each diffraction pattern. These 2D reciprocal space intensity models capture the average of aligned copies of a subset of patterns from the entire dataset, following which class averages were manually selected corresponding to single particles, indicated by high fringe contrast and a convex envelope. This procedure was then repeated, each time rejecting the various contaminants like aggregates and other outliers (details in Supplementary Section 1).

The final selection contained intensity models revealing distinctive diffraction features corresponding to an icosahedral particle with good contrast and sharp streaks. The subset of diffraction frames associated with this intensity model was selected to reconstruct a three-dimensional (3D) Fourier model using *Dragonfly*. Icosahedral symmetrization was applied due to the subset having only 7249 patterns. Subsequently, it was phased to retrieve the electron density of the MS2 capsid, as shown in Fig. 1d, with an estimated resolution of 6.1 nm (phase-retrieval parameters in the “Methods” section).

Even at this resolution, this structure is markedly different from the one obtained using cryo-electron microscopy (cryo-EM) on the same sample batch shown in Fig. 1b. This structure at 0.49 nm resolution provides insight into the conformation of the hydrated, flash-frozen capsid, which is a near-spherical particle with icosahedral symmetry (see the “Methods” section for details). This baseline structure, along with the similar crystallographic structure of the capsid (PDB: 2MS2)^35^ serves as a reference for interpreting structural changes induced by aerosolisation. The low-pass-filtered version shown in Fig. 1c shows differences at both the 5-fold and 3-fold sites, with the X-ray structure indenting inwards at the 3-fold sites.

The fact that only a limited number of patterns (only 2.5%) went into the final 3D structure with the conventional X-ray SPI analysis pipeline raises the question of the structures of the rejected particles and the source of the heterogeneity. Around two-thirds of the rest of the patterns were either from clusters or other contaminants or too weak to be useful. But that still left 10 times as many patterns as used in this reconstruction, which had near-icosahedral symmetry, but could not be merged. As the particles traverse through the low-humidity environment, the aerodynamic lens, and then the vacuum environment of the interaction region, the surrounding water envelope is continuously evaporating. We explore the possibility of whether the rejected patterns contain information about the transition from the hydrated state to the final structure depicted in Fig. 1d.

### Heterogeneity analysis workflow

**Fig. 2:**
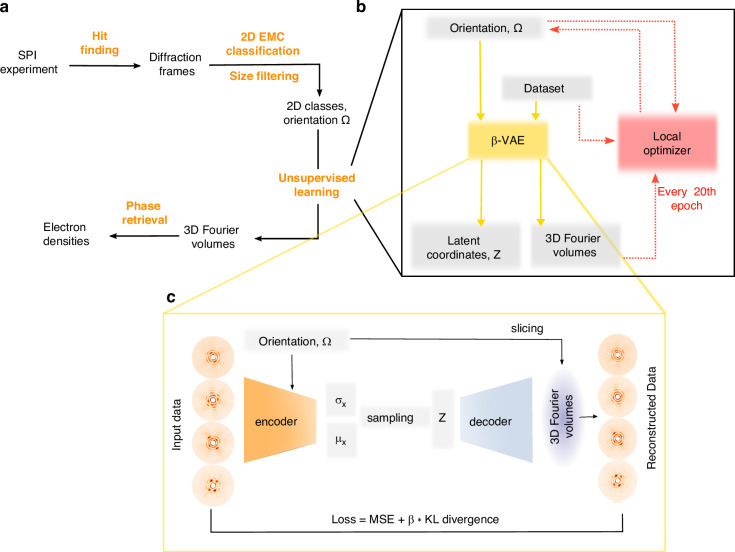
Analysis pipeline. **a** Schematic of diffraction data analysis workflow. All steps other than the unsupervised learning ones are part of the standard SPI workflow. **b** Detailed flow chart of the unsupervised learning step to generate the structural latent space and associated 3D Fourier intensity volumes. The pipeline involves training a β-VAE with a local orientation optimizer. Every 20th epoch, the optimizer outputs an improved estimate for the orientation (Ω) given the 3D Fourier volumes, dataset, and the current estimate of the orientations. **c** Schematic representation of the *β*-VAE network. The model takes 2D class-average intensities and orientations as input and encodes them into a latent space via an encoder network. This latent space coordinate (*Z*) is subsequently utilized by a decoder network to reconstruct 3D Fourier volumes

Figure 2a shows the analysis workflow for learning the structural landscape of aerosolized MS2 capsids. By using a much larger fraction of the data, we can reconstruct not just a single homogeneous object, but a whole family of structures, and then study the variations in that family. We first used the same 2D classification approach as for the single reconstruction above. In order to effectively train and utilize the deep learning method discussed below, we expanded the total number of intensity models by performing multiple runs of 2D classification. In each of the 100 independent bootstrapping runs, 20% of the diffraction frames (from a total of 170,355) were randomly selected and classified into 100 distinct 2D intensity models, resulting in 10,000 intensity models.

Upon scrutinizing the 2D intensity models, distinctive patterns emerged, including some with strong streaks in the detector plane from faceted particles but also nearly circular diffraction rings from rounded objects. These observations hinted at particle shapes spanning from icosahedral to almost perfectly round. We applied size filtering on the 2D intensity dataset to retrieve the distribution of different discrete heterogeneity in the MS2 particles. The effective size of the particles was determined from each intensity average using a spherical diffraction model^36^ (see the “Methods” section).

We curated a dataset of 2558 2D intensity models from 79,711 diffraction frames, representing particles with different capsid morphologies, but excluding models from dimers, aggregates, and other contaminants whose nominal size was outside the 23–31 nm size range. To understand the structural landscape of the remaining particles, we employed an unsupervised deep learning approach—a variational autoencoder (VAE) network^37^. Inspired by the pioneering cryoDRGN approach using a β-VAE network to study heterogeneity in single-particle cryo-EM datasets^38^ and our prior work on continuous shape transitions in gold nanoparticles^13^, we adapted the network for our MS2 virion dataset as a β-VAE, described below.

An autoencoder consists of two components, the first of which (the encoder) takes the input data and returns a low-dimensional vector, termed the latent vector. The second part (the decoder) takes this vector as input, which could even be a single number, and reconstructs the input data. Both networks are simultaneously trained to minimize the difference between the inputs and corresponding outputs. By forcing this operation to go through this bottleneck, the encoder not only learns a low-dimensional latent representation of the dataset, but the decoder can then be used to generate the data for arbitrary points in the latent space. A variational autoencoder further refines this approach by replacing the latent vector with a distribution, usually represented as the mean and standard deviation of a normal distribution. During training, instead of directly passing the estimate of the latent vector to the decoder, a random vector is sampled from the normal distribution parameterized by the encoded mean and standard deviation. The decoder has to figure out how to recover the input given a Gaussian neighborhood rather than a specific point in the latent space, which accommodates noise/uncertainties in the input and drives the smoothness of the latent representation.

In SPI experiments, not only are there unknown structural and orientation parameters, but the measurements are also incomplete, since they are 2D images of 3D objects. Thus, we implement a 2D encoder that takes 2D diffraction images, but the decoder reconstructs a 3D intensity distribution. The loss function then compares the input with a slice through this 3D intensity at the estimated orientation. In order to separate the effect of true structural variations from the large, but trivial, variations in diffraction patterns of differently oriented particles, we include the quaternion representation of the estimated orientation (Ω) for every 2D intensity model in the dense layers of the encoder network (see Fig. 2c). In the decoding process, after sampling the latent vector, the decoder reconstructs a 3D Fourier volume, which is then sliced at Ω to retrieve the input intensities as reconstructed output data. Finally, the β part of the β-VAE is a method to regularize the latent space to avoid over-fitting. This allows the network to use information from patterns in different orientations with slightly different structures. The description of the network architecture as well as the analysis workflow to refine the orientation estimates is detailed in Fig. 2b and the Supplementary Sections 2 and 3.

As an initial estimate, the orientation of each of these 2D intensity class averages was determined against a single 3D Fourier volume of the icosahedrally symmetrized MS2 bacteriophage from the conventional SPI reconstruction (Fig. 1d). These orientation estimates were incrementally updated using a so-called local optimizer, which works as follows. After a given epoch, each input data frame was used to generate a 3D Fourier volume using a single pass through the VAE. This volume was sliced multiple times, using orientations which were slightly different from the current estimate (standard deviation of 5 mrad or 0.3°). The updated orientation for this frame was chosen to be the one that maximized the Pearson correlation coefficient with the data (see Supplementary Section 3 for details). This pipeline is shown schematically in Fig. 2b.

For this dataset, the β-VAE was trained over a total of 2000 epochs. In the first 1000 epochs, the Local Optimizer was turned off, and icosahedral symmetric orientation estimates were used, allowing the VAE to learn features from the dataset and stabilize itself. In the latter 1000 epochs, the orientations were updated.

**Fig. 3:**
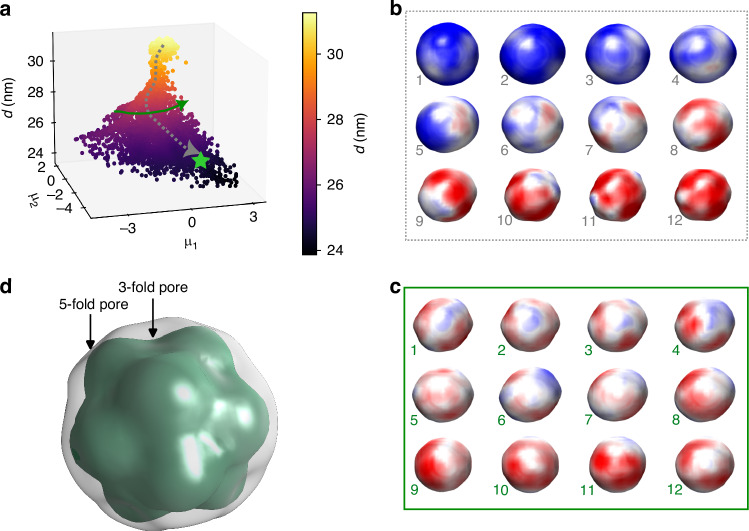
Structural landscape. **a** The latent space learned by the *β*-VAE is colored by the estimated diameter (*d*) of individual patterns. The plot highlights two distinct trajectories selected to capture the structural variation phenomenon within the latent space. The retrieved electron density of MS2 particles via phase retrieval of Fourier volumes generated by the decoder network of the β-VAE network is shown in the grid. **b** The dotted *Gray* trajectory, following from top to bottom in (a), depicts the shape-size variation in the ensemble of capsids. **c** The *Green* trajectory, progressing from left to right in (**a**), corresponds to different shape realizations for a fixed size of capsid. Red-white-blue colored radially. **d** Encapsulated overlay of the 3D structure of the MS2 capsid from the low-pass-filtered cryo-EM reconstruction (gray) and the dehydrated X-ray SPI reconstruction (green). The overlap highlights the altered conformations in the vicinity of the 5-fold and 3-fold sites

Once trained, the β-VAE network enables detailed analysis and systematic exploration of structural heterogeneity by examining the 3D intensity volumes reconstructed by the decoder for various points in the latent space. The effective diameter of each 3D volume was determined by fitting a sphere model, the result of which is shown in Fig. 3a, where the two components of the latent vector mean, *µ*_1_ and *µ*_2_, are represented along the axes, and the color and height represent the effective diameter.

### Structural landscape

The reconstructed structural landscape in Fig. 3a shows a range of sizes and structures that will be analyzed below. Prior to that, it is important to acknowledge that the landscape itself is not evidence of a temporal progression of some dynamics, since the data is effectively not timestamped. However, three observations strongly suggest that in this case, we are indeed observing snapshots of dehydration dynamics. The first is that the structure is observed using cryo-EM to be highly homogeneous in the hydrated state^39^ with an effective size of 27 nm. The second is that the initial droplets produced by the electrospray are in the range of 150–200 nm^31^. Finally, we observe particles with sizes in the range of 24–31 nm. If these images do not capture dehydration dynamics, this would imply that an initially homogeneous population of hydrated structures responds to the aerosolization process by transforming to a broad range of structures, some of which are larger than the original virus. We believe this explanation to be unlikely and will assume dehydration in the rest of this paper, while acknowledging that we cannot rule out the alternative.

We highlight two paths through the structural landscape shown in Fig. 3a, capturing two salient features of the evolution of the capsid morphology. Firstly, the dotted *Gray* line trajectory illustrates the variation in shape and size, as observed from top to bottom, which we ascribe to the effect of dehydration. Three-dimensional structures of the MS2 capsids along this path are depicted in Fig. 3b. Following this path, we note a transition from larger particles to smaller, nearly icosahedral particles as dehydration progresses. The largest particles (29–31 nm) were nearly spherical and larger than the reported 27 nm hydrated structure, representing MS2 capsids with a water envelope around them. The contour images in Fig. 3b and c are color-coded radially from red to white to blue in order to ease visualization of facets, curvature, and size changes. The structure in Fig. 1d, reconstructed without the VAE, lies at the end of this path and is highlighted by a star in the landscape.

The second, *Green*, trajectory is shown in Fig. 3c. Along this path, all particles had an estimated diameter of 27 nm. Here, subtle and gradual deviations from the icosahedral shape at a constant size are observed. Close examination shows structures with varying degrees of deviation from the symmetric structure, also borne out by individual diffraction patterns and class averages showing asymmetric structures. This suggests that whatever morphological change is occurring is not a coherent change acting on all icosahedral sites simultaneously, but seems to occur independently at each site.

### Proposed molecular mechanism

In order to better understand the capsid morphology change, we focus on the fully dehydrated state and compare it to the MS2 capsid structures obtained from cryo-EM and crystallography. Figure 3d displays an overlay visualization of the two MS2 capsid structures: the low-pass filtered cryo-EM reconstruction and the X-ray SPI reconstruction. This overlay emphasizes the locations of the pores at the 5-fold and 3-fold sites (vertex and face center, respectively), which are affected during dehydration through aerosolisation. The *T* = 3 icosahedral capsid of MS2 consists of 12 5-fold contacts at the vertices and 20 6-fold contacts at the face-centers, as seen in the cryo-EM structures in Fig. 1b and c. The configuration of the coat protein creates a capsid shell featuring 32 pores (about 2 nm in diameter), denoted here as 5-fold and 3-fold pores, respectively.

The crystal structure of the MS2 virus capsid^35^ shows that the coat protein has three possible conformations, termed A, B, and C. These proteins assemble into two types of dimers: asymmetric A/B dimers and symmetric C/C dimers. Although the A, B, and C subunits (129 residues) are almost structurally identical, they differ in the conformation of the FG-loop (residues 66-82), with the A and C subunits exhibiting a conformation that is different from that of the B subunit.

**Fig. 4:**
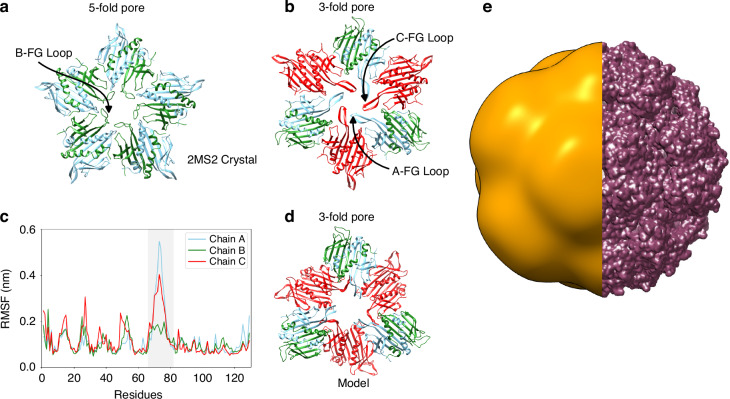
Dehydrated capsid model. The pentameric (5-A/B) and hexameric (3-A/B and 3-C/C) faces of the *T* = 3 icosahedral capsid shell from the 2MS2 PDB structure. At the 5-fold axis, FG loops of B (B-FG loop, green, **a**), and at the 3-fold axis, FG loops of A (A-FG loop, sky blue, **b**) and C (C-FG loop, red) are crucial for capsid assembly and curvature. **c** The A-FG loop and C-FG loop exhibit significant fluctuations compared to the B-FG loop (residues 66-82, shaded region). The root mean square fluctuation (RMSF) was calculated from a 20 ns vacuum MD-trajectory of A/B and C/C dimers. **d** Transformed hexameric building block designed/modeled from the X-ray SPI map. At the 3-fold axis, C/C dimers move toward the capsid center. **e** Map generated from transformed capsid model (at 6.1 nm resolution). The left half is in a similar representation as the experimental X-ray SPI map (Fig. 1d) for visual comparison

The 5-fold pores consist of 5 A/B dimers, with the FG-loops of the five B-subunits oriented towards the pores in a compact conformation, as depicted in Fig. 4a. The 3-fold pores are formed by six dimers—3 A/B and 3 C/C—arranged alternately, with 3 FG-loops from each A and C subunit in an extended conformation (Fig. 4b). The FG-loop plays a pivotal role in capsid assembly and affects its curvature and mutations in this region can disrupt assembly^40^.

We performed molecular dynamics (MD) simulations of the A/B and C/C dimers in vacuum conditions similar to those during sample delivery of the SPI experiment (see the “Methods” section for details). The FG-loop of A and C subunits showed notable conformational changes or movements compared to the FG-loop of B on a nanosecond timescale (marked in gray in Fig. 4c). The dehydration primarily affects the FG-loop of A and C^41,42^, suggesting a strong role for water molecules in stabilizing the extended form of the FG-loop around the 3-fold pore. In addition, mass spectrometry observations hint that a section of the internal RNA stabilizes the A/B dimers of the capsid^43^.

Based on these observations, we formulate a hypothesis that due to the high mobility of the FG-loops of A and C under dehydrating conditions, the FG-loops around the 3-fold pore contract upon losing stabilizing waters, and the C/C dimer shifts towards the center of the virus. We utilized the positions of A, B, and C subunits from the asymmetric unit of the 2MS2 crystallographic model as a starting point, then adjusted the position of the C subunit (by translation and rotation) to form a new capsid assembly and minimized the energy of the entire capsid model in vacuum conditions. This procedure was iterated until we obtained a stable capsid shell model, which also fit our SPI electron density map, shown in Fig. 4d. Figure 4e shows the full capsid with the modeled pore structure. The left half shows the low-pass-filtered electron density map, showing a remarkable similarity to the experimental map in Fig. 1d.

## Discussion

The structural response of viruses to a dehydrating environment is an important and somewhat understudied question, limited by the inability to study these systems in situ under these conditions. Single-particle imaging using XFELs provides a unique opportunity to probe the structures of these viral capsids while they are dehydrating in an aerosol stream. With the use of machine learning tools to classify the whole ensemble of observed particles, one can observe complex conformational trajectories that would be hidden with other ensemble-averaged measurements. The femtosecond XFEL pulses allow one to temporally freeze the structural transitions and observe non-equilibrium, intermediate structures that occur during dehydration.

In this work, we apply this method to MS2 bacteriophage capsids, where we observe 3D structures ranging from a well-hydrated particle with a liquid envelope down to a dehydrated structure, with a different capsid morphology. Not only do we see these endpoints, but also a large number of intermediate conformations which break icosahedral symmetry, providing clear evidence for a site-specific transformation rather than a capsid-wide concerted change. Some open questions remain, the first being how general these dynamics are for other viruses with different capsid symmetries. It also remains to be explored how aerosolized viruses at room temperature and ambient pressure respond. The success of native mass spectrometry studies under harsher conditions than applied in this work suggests that these dynamics may not be wholly foreign^44^. Finally, as discussed in the Results Section, further validation is required to conclusively establish the temporal nature of the observed structural heterogeneity. Some approaches to achieve this would be to perform SPI with varying initial droplet diameters and controlled humidity, but also using time-resolved AFM.

While this study is limited to a moderate resolution, the large-scale changes in this system are already clearly apparent. Background scattering is the primary barrier to achieving sub-nm resolution using X-ray single-particle imaging. Two avenues of technical improvements show promise in increasing the achievable resolution, involving either reducing the background using helium-based aerosol delivery^45^ or increasing the tolerance to background using strongly scattering holographic references^46^. This work also opens up the possibility of studying this important question for aerosol-transmitted pathogenic viruses.

## Materials and methods

### Sample preparation

*E. coli* strain C-3000 (*ATCC 15597*) was cultured in volumes of 50 mL at 37 °C with shaking at 150 rpm. Shaking was reduced to 90 rpm when the exponential growth phase was reached, and the culture was infected with 100 µL MS2 (2.9 mg mL^−1^, *ε*_280_ = 3.86 mg mL^−1^) (*ATCC 15597-B1*) and 100 µL CaCl_2_ (1 M). Incubation was stopped when the cells were lysed (ca. 3 h). One milliliter of the lysate and 800 µL CaCl_2_ were used to infect 400 mL of exponential phase growth culture of *E. coli*. Incubation was carried out with shaking at 90 rpm until the cells were lysed (ca. 5 h). The lysate was precipitated using 10% (w/v) PEG 6000 and 0.5 M NaCl over 48 h at 4 °C. After precipitation, the suspension was centrifuged at 10,000×*g* for 30 min. The pellet was re-suspended in 30 mL 0.01 M Tris, pH 7.5 (containing 0.1 M NaCl, 0.1 mM MgCl_2_, and 0.01 mM EDTA). Stirring was carried out for 1 h at room temperature until complete re-suspension. Next, the suspension was incubated at 37 °C with shaking at 120 rpm after adding 1.5 mg lysozyme, 300 µL MgCl_2_ (1 M), and 10 µL Benzonase. After incubation, the suspension was centrifuged at 8000×*g* for 30 min. The supernatant was precipitated using 10% (w/v) PEG 6000 and 0.5 M NaCl and incubated at 4 °C overnight. The suspension was centrifuged at 27,000×*g* for 30 min, and the pellet was re-suspended in Tris buffer. The re-suspension was applied to a sucrose gradient (15–50%) and centrifuged at 40,000×*g* for 18 h at 4 °C. The sucrose in the collected band fractions was removed by repetitive concentration and dilution steps with Tris buffer using an Amicon Ultra Centrifugal Filter (100 kDa cutoff). Prior to cryo-EM grid preparation and sample injection at the XFEL, the Tris buffer of the sample was exchanged to a buffer containing 0.2 mM sodium citrate and 5 mM ammonium acetate using a PD Minitrap G-25 column (Cytiva). The sample concentration was adjusted to ∼2 × 10^15^ particles/mL (or ∼12 mg/mL) for both experiments.

### Cryo-EM structure determination

An aliquot (3 µL) of MS2 virions was deposited onto freshly glow-discharged, 300 mesh R2/2 Quantifoil grids, followed by 3 s of blotting at 4 °C and 95% humidity using a Vitrobot Mark IV instrument (ThermoFisher Scientific). The blotted grid was plunge-frozen into a 37:63 (v/v) liquid ethane/propane mixture. Images were acquired using a Talos Arctica microscope (ThermoFisher Scientific) operated at 200 kV and equipped with a Falcon 3EC detector (ThermoFisher Scientific). A total of 861 movies were recorded using the EPU software (ThermoFisher Scientific) in integration mode at a nominal magnification of ×92,000, yielding a final pixel size of 1.58 Å^2^. Each movie had a total dose of 36e^−^/Å^2^ over 39 frames. Image processing was performed using cryoSPARC^47^. Drift and beam-induced motions were corrected using patch motion correction, and the contrast transfer function (CTF) was estimated using patch CTF estimation. The micrographs were inspected and curated using the manually curated exposures job, from which 622 micrographs were accepted for further processing. Blob picking was used to pick 60,861 particles, of which 47,546 remained after two rounds of 2D classification. Two classes out of four from ab initio reconstruction and heterogeneous refinement (C1 symmetry) had apparent density for both the capsid and the A protein. The particles from these two classes (22,592) were selected for homogenous refinement (C1), where a 4.9 Å resolution map was obtained as estimated by the Fourier shell correlation (FSC) = 0.143 criterion (see Fig. S1).

### SPI data collection

Data was collected at the SPB/SFX (single particles, clusters and biomolecules & serial femtosecond crystallography) instrument^48^ of the European XFEL using 6 keV photons focused into a 250 × 250 nm^2^ spot. Individual X-ray pulses were generated with 3.8 mJ of energy on average (3.94 × 10^12^ photons/pulse). The pulses were delivered in 352-pulse trains with an intra-train repetition rate of 1.1 MHz and trains arriving every 0.1 s, leading to a maximum data collection rate of 3520 frames/s. A detector built specifically for this burst mode operation, the adaptive gain integrating pixel detector (AGIPD)^49^, was placed 700 mm downstream of the interaction region to collect the diffraction patterns for each pulse individually up to a scattering angle of 13° at the corner of the detector.

### MD simulation

We employed the Gromacs package^50^ for our simulations, utilizing the OPLS-AA force field^41^ to investigate the A/B and C/C dimers in vacuum conditions. The initial configurations were based on the 2MS2 PDB structure^35^. To achieve a total charge of +10e for the dimers, we protonated specific aspartic and glutamic acid residues within each subunit^43^, adhering to a well-established protocol^41^. Subsequently, the structures underwent a steepest descent energy minimization followed by a brief equilibration at 300 K, without the application of periodic boundary conditions or pressure coupling, to simulate vacuum conditions. Protein dynamics were monitored over a 20 ns period, with all parameters maintained in alignment with the established protocol^41^.

### Phase retrieval

The electron densities were reconstructed through a 3D iterative phase-retrieval method applied to the full-resolution intensity volume of the MS2 bacteriophage. The procedure was almost identical to the pipeline discussed in the ref. ^16^. Figure 1d illustrates the reconstructed electron density obtained for a dehydrated phage. In Fig. S4, the phase retrieval transfer function (PRTF) metric, evaluating the reproducibility of retrieved phases based on 128 independent phasing runs for both icosahedral and octahedral structures of MS2 capsid. The electron density reconstruction from the background-subtracted intensity distribution involved a hybrid approach employing the error reduction (ER) algorithm and the difference map (DM) algorithm. Each phasing run consisted of 400 iterations, comprising 100 ER iterations followed by 200 DM iterations, and concluding with 100 additional ER iterations. The support was updated after each iteration using a smoothing and thresholding procedure, with the strongest 40,000 voxels retained in the support. The phase-retrieval process for the reconstructed Fourier volumes by the decoder network for various trajectory points involved 16 random model starts. The number of voxels for the support was determined based on the estimated diameter size and fringe counts in the 3D Fourier volumes. The electron density maps were visualized with radial coloring to depict structural variations. The density map, crystal structure, and SPI densities were visualized using the Chimera software^51^.

### Particle size determination

We utilized spherical particle fitting on the Fourier volumes reconstructed by the decoder network of *β*-VAE. This process involved computing the radial average of the volumes and fitting them with the Fourier model of a spherical particle. This analysis yielded an estimation of the diameter of the MS2 phages during the shape-phase transition. The Fourier model for a spherical particle is described by the function *S*(*q, d*):

$$S(q,d)\propto {d}^{6}\left(\frac{\sin (\pi qd)-\pi qd \cdot \cos(\pi qd)}{(\pi qd)^3}\right)^2$$

where *d* denotes the diameter of the particle and *q* is defined with the crystallography convention. The size distribution of MS2 bacteriophage is shown in Fig. S7.

## Supplementary information

Supplementary Information

## Data Availability

The raw data is available here: 10.22003/XFEL.EU-DATA-002734-00.

## Code availability

The code for the β-VAE is available at https://github.com/AyyerLab/StrucNN/tree/master/ms2vae. The *Dragonfly* source code is available at https://github.com/duaneloh/Dragonfly.
