# Supplementary material for: High-throughput in situ single particle X-ray imaging of dehydrating viral capsids
Source: Light Sci Appl. 2026 Jun 23;15:280. doi: 10.1038/s41377-026-02262-0 (PMC13291355; doi:10.1038/s41377-026-02262-0)
Supplement: Supplementary file 1 — Supplementary Information [file 41377_2026_2262_MOESM1_ESM.pdf]

Supplementary Information for: High-throughput *in situ* Single Particle X-ray Imaging of Dehydrating Viral Capsids

Abhishek Mall,[1] Anna Munke,[2,3] Zhou Shen,[1] Parichita Mazumder,[1] Johan Bielecki,[4] Juncheng E,[4] Armando Estillore,[2] Chan Kim,[4] Romain Letrun,[4] Jannik Lübke,[2] Safi Rafie-Zinedine,[4,5] Adam Round,[4] Ekaterina Round,[4] Michael Rütten,[6] Amit K. Samanta,[2,7] Abhisakh Sarma,[4] Tokushi Sato,[4] Florian Schulz,[6] Carolin Seuring,[8] Tamme Wollweber,[1,7,9] Lena Worbs,[2,9] Patrik Vagovic,[4] Richard Bean,[4] Adrian P. Mancuso,[4,10,11] Ne-Te Duane Loh,[12] Tobias Beck,[6] Jochen Küpper,[2,7,9,13] Filipe R.N.C. Maia,[3,14] Henry N. Chapman,[2,9,7] and Kartik Ayyer[1,7,*]

[1] *Max Planck Institute for the Structure and Dynamics of Matter, 22761 Hamburg, Germany*
[2] *Center for Free Electron Laser Science, Deutsches Elektronen Synchrotron (DESY), 22607 Hamburg, Germany*
[3] *Laboratory of Molecular Biophysics, Department of Cell and Molecular Biology, Uppsala University, Uppsala, SE-75124, Sweden*
[4] *European XFEL, Holzkoppel 4, 22869 Schenefeld, Germany*
[5] *Institute of Biomaterials and Biomolecular Systems, University of Stuttgart, Pfaffenwaldring 57, 70569 Stuttgart, Germany*
[6] *Universität Hamburg, Institute of Physical Chemistry, Grindelallee 117 20146 Hamburg, Germany*
[7] *The Hamburg Center for Ultrafast Imaging, Universität Hamburg, 22761 Hamburg, Germany*
[8] *Centre for Structural Systems Biology (CSSB), Notkestraße 85, 22607 Hamburg, Germany*
[9] *Department of Physics, Universität Hamburg, Luruper Chaussee 149, 22761 Hamburg, Germany*
[10] *Department of Chemistry and Physics, La Trobe Institute for Molecular Science, La Trobe University, Melbourne, VIC, 3086, Australia*
[11] *Diamond Light Source, Harwell Science and Innovation Campus, Didcot, Oxfordshire, OX11 0DE, UK*
[12] *National University of Singapore (NUS), Dep. of Physics / Fac. of Science , 2 Science Drive 3 Singapore 117542 , Singapore*
[13] *Department of Chemistry, Universität Hamburg, 20146 Hamburg, Germany*
[14] *NERSC, Lawrence Berkeley National Laboratory, Berkeley, CA, 94720, United States*

[*] kartik.ayyer@mpsd.mpg.de

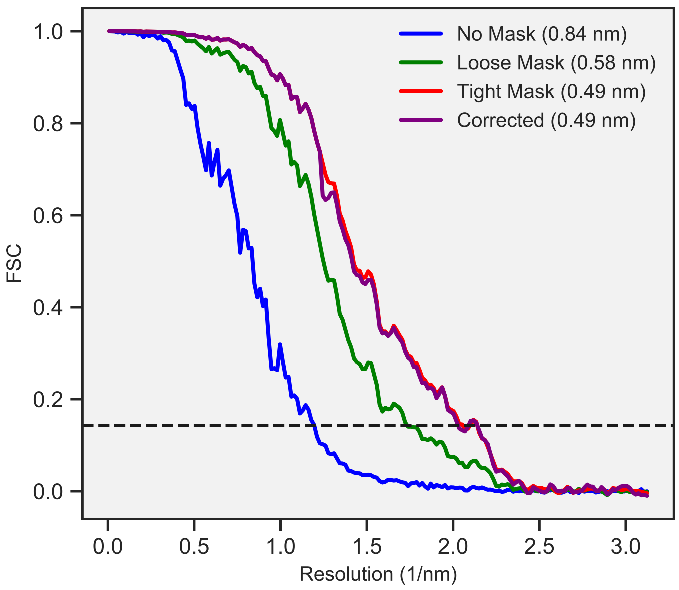

FIG. S1. FSC curve of the cryo-EM reconstruction generated using cryoSPARC [1] for the cryo-EM reconstruction of the MS2 capsid from the same batch as used for the X-ray SPI experiment. The average resolution of 0.49 nm was estimated based on FSC = 0.143 threshold [2] (black dashed line).

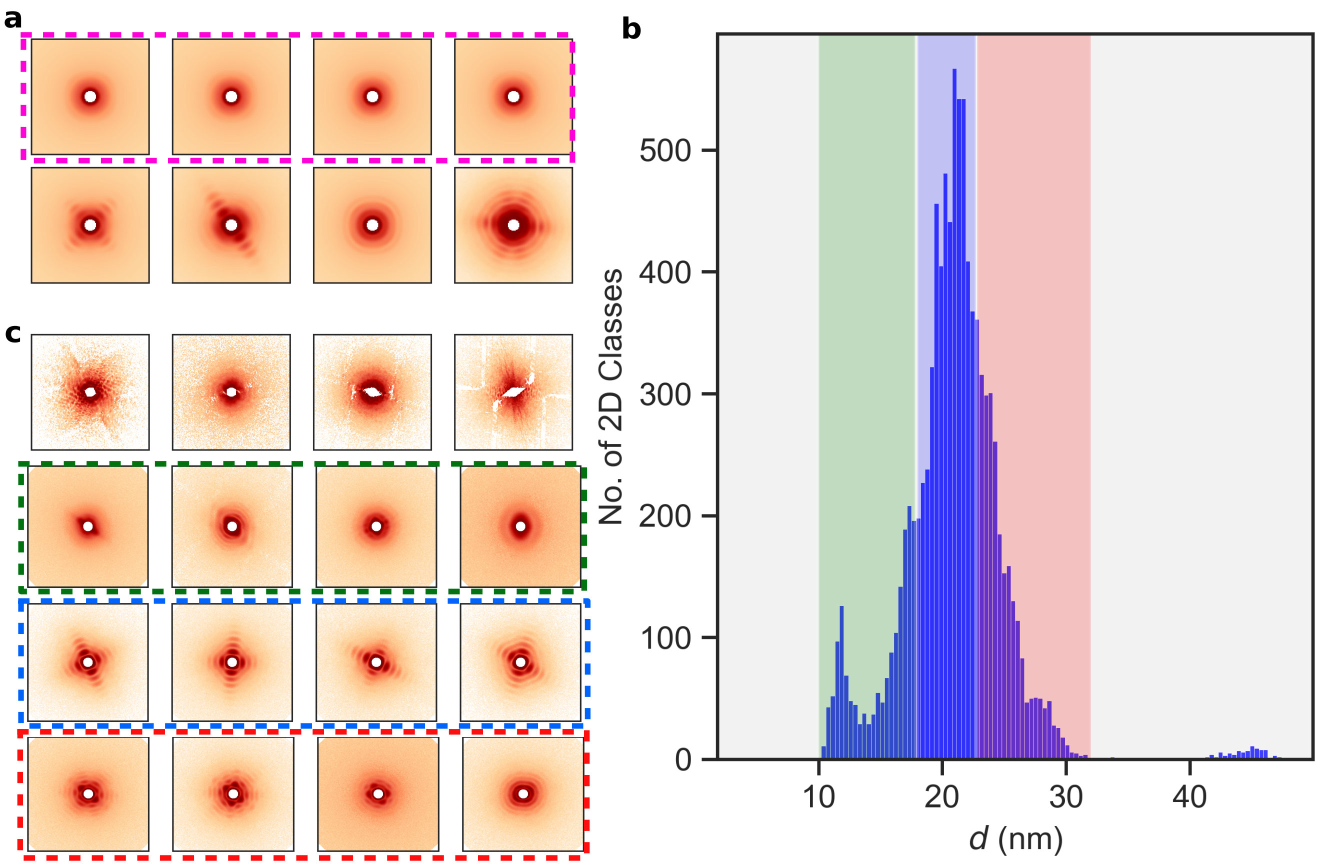

FIG. S2. (a) Examples from the 50 2D intensity models obtained from the initial EMC classification. (Dashed pink grid) Models rejected prior to generating the training dataset for the β-VAE. (Bottom row) Examples of the 2D intensity models used for the dataset generation. (b) Histogram of the fitted diameters for the 2D intensity classes dataset (10 000 classes). Different structure types are manually marked in the distribution as icosahedral (red), octahedral (blue), and others, including dimers, outliers, etc. (green). (c) The corresponding examples of 2D intensity models for each structure class. (Top row) Examples of classes with panel gaps and detector artifacts, which had fitted diameters > 40 nm.

1. CLASSIFICATION, DISCRETE HETEROGENEITY AND POLYMORPHISM

1.1. Classification

The first step of data classification was the generation of average two-dimensional (2D) classes in the detector plane from the full diffraction dataset using the 2D classification procedure implemented in Dragonfly [3, 4]. This process employs a modified EMC algorithm to classify all frames into a specified number of averages (models, termed classes in Dragonfly). We began by classifying the dataset into 50 2D classes. Examples of these classes are shown in Fig. S2a. The 2D classes corresponding to very weak hits (pink dashed grid) were excluded at this stage.

To obtain the training dataset, a bootstrapping method was employed by running the 2D EMC reconstruction 100 times, each with 100 models, using a random subset of 20% of the frames (from 170 355 diffraction frames) each time, resulting in 10 000 2D intensity models. Size filtering was then applied to the dataset by fitting a spherical object Fourier model to the radial average of the intensity, resulting in a size distribution of 2D intensity models (Fig. S2b). By comparing the 2D models and their locations in the distribution, we qualitatively divided the space into three groups, as shown in Fig. S2b. In the figure, red denotes icosahedral, blue denotes octahedral, and green denotes contaminants, including outliers and dimers. The corresponding example samples of 2D intensities for different groups are shown in Fig. S2c. The top row shows the classes with panel gaps and detector artefacts, which have fitted diameters greater than 40 nm in the distribution. Among all dataset models, 2558 were icosahedral corresponding to 79 771 diffraction frames. These icosahedral 2D intensity models were used for training the β-VAE.

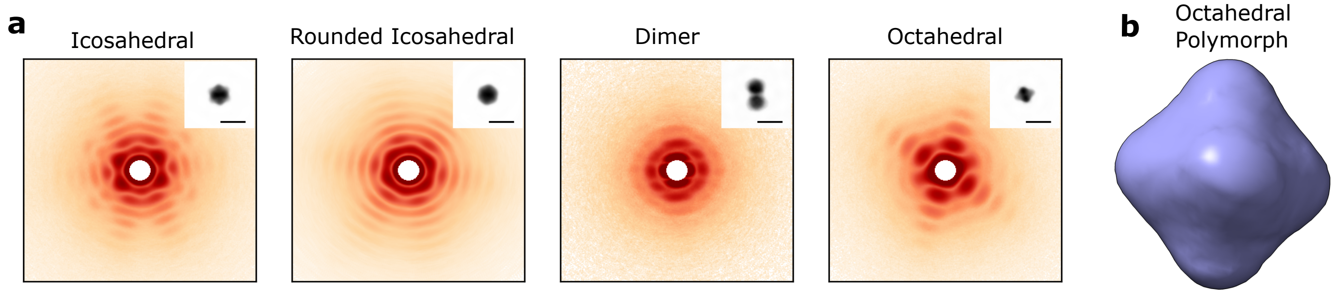

FIG. S3. (a) Examples from the diffraction dataset of 2D intensity models and corresponding electron density projections (inset) via phase retrieval; the scale bar is 30 nm. (b) 3D structure of the MS2 capsid reconstructed from the octahedral data.

1.2. Discrete Heterogeneity

The 2D classification also yielded some interesting structures which had a different symmetry than the icosahedral objects. Figure S3a shows some of the 2D intensity averages with reasonable intensity contrast. Note that since intensities are always non-negative, the averages from diverse aggregates and contaminants typically generate low-contrast models. The insets show the projected electron densities resulting from 2D phase retrieval. Only patterns belonging to classes like the "Icosahedral" class were selected for Fig. 1d.

1.3. Polymorphism

Along with the rounded icosahedra and dimers, we also obtain patterns with clear octahedral structure. The 3D structure of the octahedral particle was reconstructed without any imposed symmetry from 11 626 patterns. The reconstructed electron density at 6.1 nm resolution is shown in Fig. S3b and is 1.53 times lower in volume than the icosahedral structure. MS2 capsids have been reported to assemble with octahedral packing and $T = 3$ quasi-symmetry [5, 6]. The primary distinction between icosahedral and octahedral structures lies in the presence of four-fold contacts rather than five-fold contacts, potentially resulting in curved interfaces. The octahedral packing results from the fusion of two coat-protein subunits [7]. These capsids have been reported to disassemble and reassemble into the octahedral structure during crystallisation [5]. To the best of our knowledge, this is the first observation of octahedral MS2 capsids without mutation or different buffer conditions.

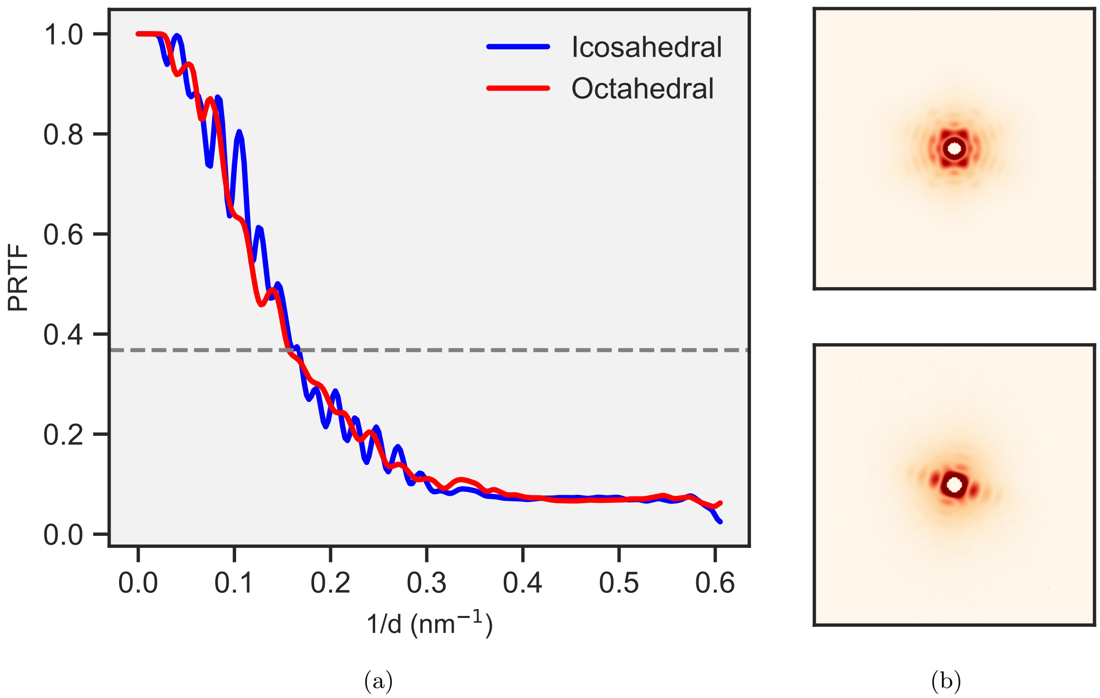

FIG. S4. (a) Smoothed phase retrieval transfer function (PRTF) vs q. The solid lines represent the azimuthal average PRTF conventionally used to determine the resolution of the structure. The typical $1/e$ cutoff is shown in gray. The resolution at the cutoff for both capsid structures was estimated to be ≈ 6.1 nm. (b) Slice at 001-plane through the Fourier volume of the MS2 capsid for icosahedral (top) and octahedral (bottom) structures retrieved using *Dragonfly* [3]. Fig. 1d & Fig. S3b shows the corresponding phased electron density.

2. ARCHITECTURE AND TRAINING OF β-VAE

The β-VAE consisted of an *encoder* and a *decoder* neural network, to encode information into a lower dimension and retrieve it back respectively. The encoder encodes diffraction data (in this case, 2D intensity models) generating a low-dimensional latent vector, Z, for each input pattern $\mathbf{X}$. The encoder parameterises this distribution with a mean $\mu(\mathbf{X})$ and a variance $\sigma(\mathbf{X})$. During training, this distribution is sampled from a normal distribution $\mathcal{N}(\mu(\mathbf{X}), \sigma(\mathbf{X}))$ before being passed to the decoder, which introduces stochasticity, improving robustness and ensuring smoothness of the latent space.

The network was trained and optimised by minimising a loss function, combining mean square error as a reconstruction loss and Kullback-Leibler (KL) divergence loss as a regularization term, which discourages a too-sharp latent space. In our case, the optimized β-VAE had $\beta = 0.5$, with the latent space dimension of $Z = 2$.

2.1. Pre-processing

The initial 2D intensities from Dragonfly have dimensions of 503 $\times$ 503 pixels. Preprocessing steps were applied to enhance relevant features and reduce computational redundancy. Given the highly sampled nature of the data and minimal scattering signal at high q, the size was reduced to 171 $\times$ 171 through downsampling and cropping. Additionally, background normalisation was performed by subtracting the mean at high q and dividing by the mean at low q. Considering that diffraction patterns of compactly supported objects are primarily dominated by low q signal, to appropriately weight higher q shape information, the 2D intensities were divided by the radial average intensity over the whole dataset before inputting them into the network. This weighting was then reverted when generating the 3D Fourier volumes. This approach optimises computational efficiency by focusing solely on relevant information in the diffraction data, where distinctive features are evident.

2.2. Network Parameters

The encoder network consists of a series of convolutional layers, specifically three Conv2d layers that increase in channel depth from 8 to 32, followed by a sequence of linear layers reducing the dimensionality to a latent space dimension Z. Conversely, the decoder utilizes a symmetrical setup starting from the latent dimension Z, expanding through linear layers, and then upscaling spatial dimensions through three ConvTranspose3d layers, ultimately reconstructing the input data. Other optimized hyperparameters of the β-VAE include a batch size of 32 and a learning rate of 10^{-4} and wight decay of 10^{-5} for Adam optimizer [8]. The architecture parameters of the β-VAE are detailed in Table I.

2.3. Training performance

The β-VAE was trained over a total of 2000 epochs. Fig. S5(a) depicts the generative performance of the β-VAE on 2D intensity data at the final epoch. The majority of prominent features are successfully reconstructed in the output, indicating high-quality reconstruction performance. This highlights the effectiveness of the deep learning model in capturing the fundamental attributes inherent in the input data.

Fig. S5(b) illustrates the loss of the β-VAE over the final 1000 epochs, during which the orientation was updated every 20th epoch before terminating the training. This approach was adopted because the loss stabilized with no significant changes observed. These stable training dynamics suggest efficient convergence of the VAE and optimization of orientation estimates for each 2D intensity model.

2.4. Choosing β and latent space dimensions

Figure S5(c) illustrates the VAE training to determine the optimal value of β. The process involved training multiple VAE networks across a range of β values from 0 to 10. The optimal value was chosen based on achieving the minimal loss. The selection of $\beta = 0.5$ strikes a balance between smooth disentanglement in the latent space and preservation of reconstruction quality, providing sufficient regularization to prevent overfitting.

Similarly, for latent space dimension $Z > 2$, there was a reduction in MSE loss; however, this improvement did not reveal any new or distinctive features in the latent space. Conversely, $Z = 2$ seemed to effectively encapsulate the variations in the dataset.

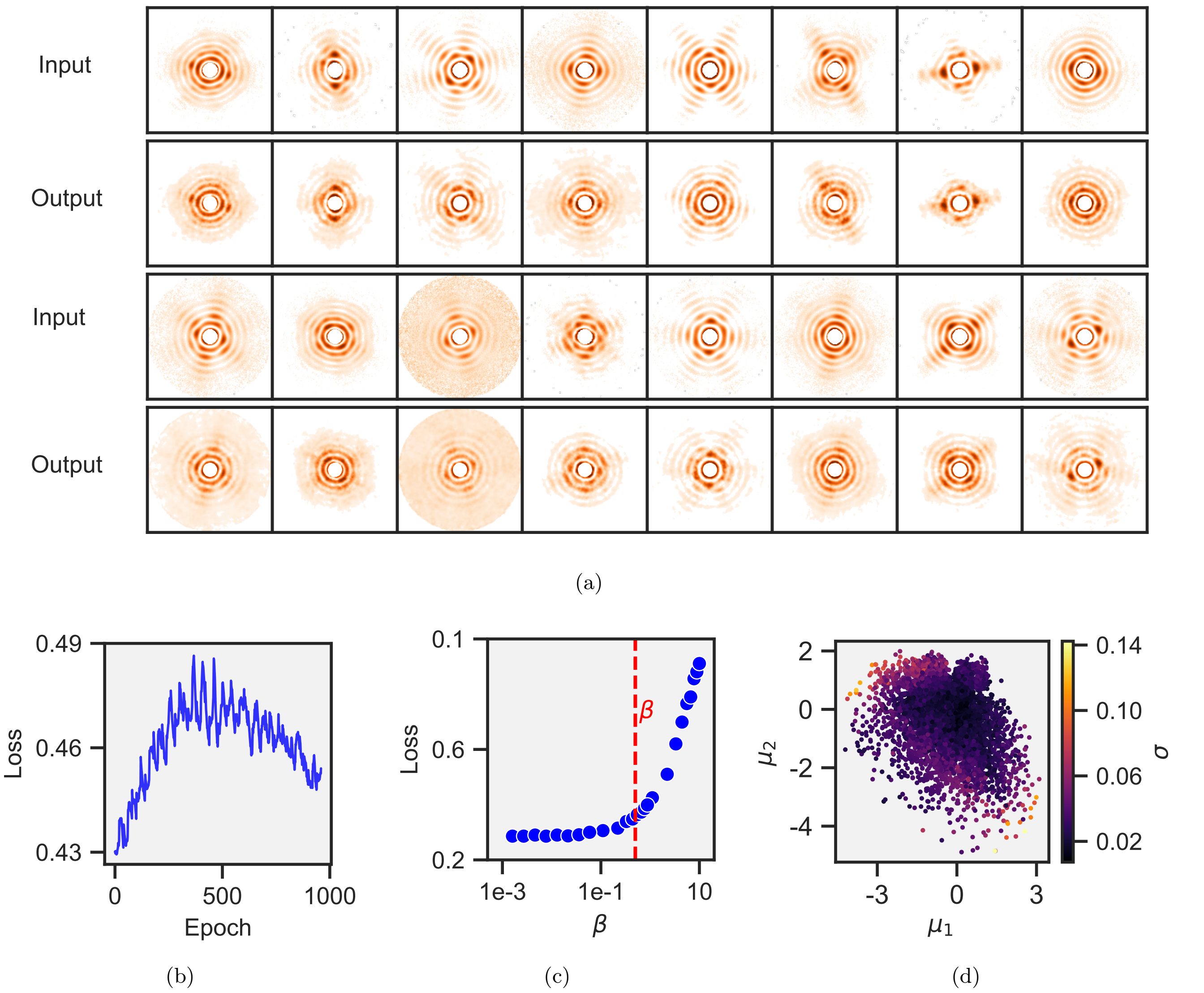

FIG. S5. (a) Comparison between input 2D intensity data and the corresponding output (reconstruction) by β-VAE. (b) Loss evolution during β-VAE training exhibited a decline over the 1000 epochs. However, the decrease was not significant later in training, prompting the decision to terminate further training. The depicted loss encompasses both the Mean Squared Error (MSE) loss for reconstruction and the Kullback-Leibler (KL) divergence loss. (c) β-VAE Loss *versus* β values. The plot illustrates a rise in loss as β values increase. The optimal trade-off between minimizing loss and providing sufficient regularization occurred at $\beta = 0.5$ (red dashed line). (d) Latent space representation of the β-VAE color labeled with $\sigma = \sqrt{\sigma_1^2 + \sigma_2^2}$.

Figure S5(d) shows the latent space representation color-coded by $\sigma = \sqrt{\sigma_1^2 + \sigma_2^2}$. The low standard deviation values suggest that the network can effectively extract and learn significant features, which are closely correlated and can be accurately reconstructed with minimal uncertainty.

2.5. Stability for different random initialization

Figure S6 shows the latent space representation of the β-VAE for three different random initializations of weights and biases. Specifically, random seeds of 42, 61, and 99 were used, respectively. Although the low-dimensional embeddings appear different due to these random initializations, the latent space consistently captures similar information regarding shape and size variation across all three cases. This demonstrates the robustness of the β-VAE in retrieving information from diffraction data despite variations in initial conditions.

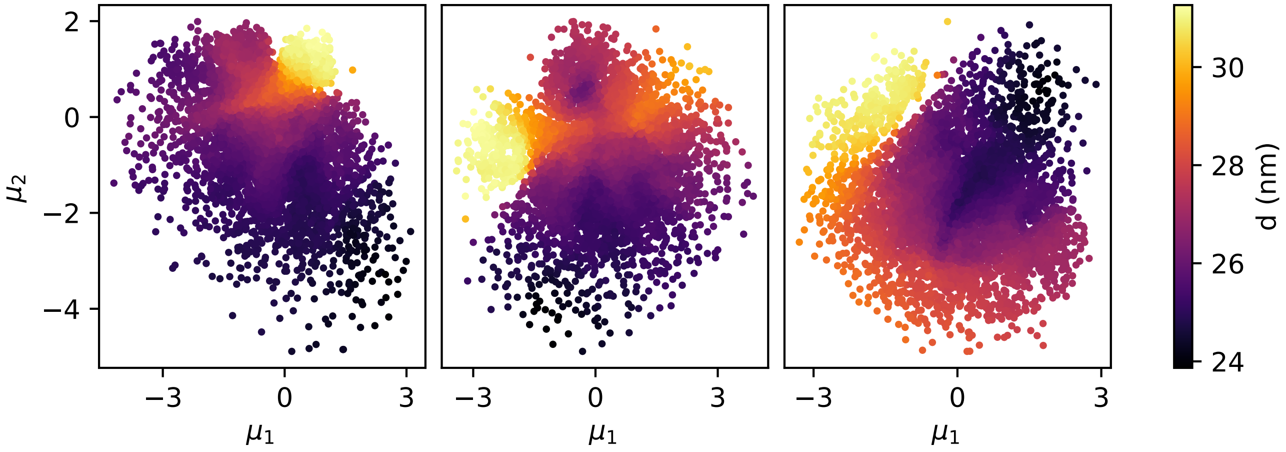

FIG. S6. Latent space representation of the β-VAE for different random initializations of weight and bias parameters. The low-dimensional embedding varies due to the random start, converging to nearby minima. However, it maintains the same information for shape and size variations.

**Network**	**Layer**	**Output Size**	**Weights**	**Bias**
Encoder	Conv2d (1, 8)	$H/3 \times W/3 \times 8$	$5 \times 5 \times 1 \times 8$	8
	Conv2d (8, 16)	$H/9 \times W/9 \times 16$	$5 \times 5 \times 8 \times 16$	16
	Conv2d (16, 32)	$H/27 \times W/27 \times 32$	$5 \times 5 \times 16 \times 32$	32
	Linear	128	800×128	128
	Linear	64	128×64	64
	Linear	8	64×8	8
	Linear (mean)	Z	$8 \times Z$	Z
	Linear (log variance)	Z	$8 \times Z$	Z
Decoder	Linear	64	$Z \times 64$	64
	Linear	$128 \times 5 \times 5 \times 5$	$64 \times 128 \times 5 \times 5 \times 5$	128
	ConvTranspose3d (128, 64)	$H/3 \times W/3 \times D/3 \times 64$	$5 \times 5 \times 5 \times 128 \times 64$	0
	ConvTranspose3d (64, 32)	$H \times W \times D \times 32$	$5 \times 5 \times 5 \times 64 \times 32$	0
	ConvTranspose3d (32, 1)	$H \times W \times D \times 1$	$7 \times 7 \times 7 \times 32 \times 1$	0

TABLE I. Architecture of β-VAE

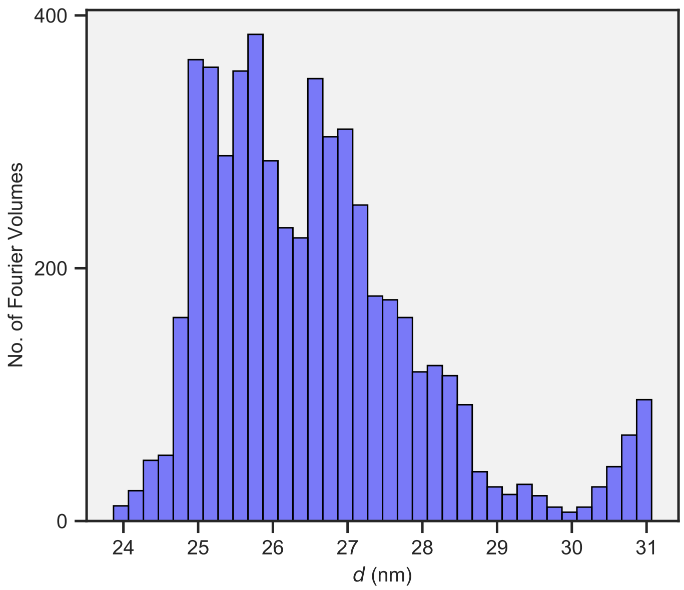

FIG. S7. Histogram depicting the particle size distribution, represented by fitted diameter values, d (nm). These values were obtained through spherical particle model fitting on the Fourier volumes reconstructed using the *decoder* network of the optimized β-VAE.

3. LOCAL ORIENTATION OPTIMIZER

The Local optimizer updates the orientation (Ω) every 20th epoch during training. To monitor the convergence of orientation estimates, we assess the Root Mean Square Deviation (RMSD) between estimates at consecutive update steps (Fig. S8). Convergence is quantified by the measured angle, denoted as Θ, between orientations represented by quaternions at consecutive update steps. Θ is calculated as

$$\Theta = \arccos\left(2 \cdot (\mathbf{q}_1 \cdot \mathbf{q}_2)^2 - 1\right) \tag{1}$$

where $\mathbf{q}_1$ and $\mathbf{q}_2$ are the normalized quaternions representing orientations.

The RMSD is computed over these angles to measure the average deviation between orientations across update steps of the Local Optimizer. It is determined as

$$\text{RMSD} = \sqrt{\frac{1}{n}\sum_{i=1}^{n}\Theta_i^2} \tag{2}$$

where n is the number of data samples and Θ_i is the angle between orientations for data sample i at two consecutive epochs.

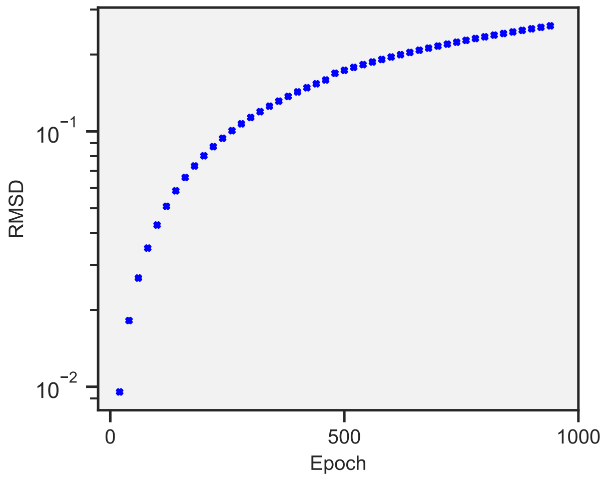

FIG. S8. Root mean square deviation (RMSD) *vs* Epoch. The RMSD values were evaluated between the orientation estimates at two consecutive updates of the Local Optimizer. The update is performed every 20th epoch of the training.

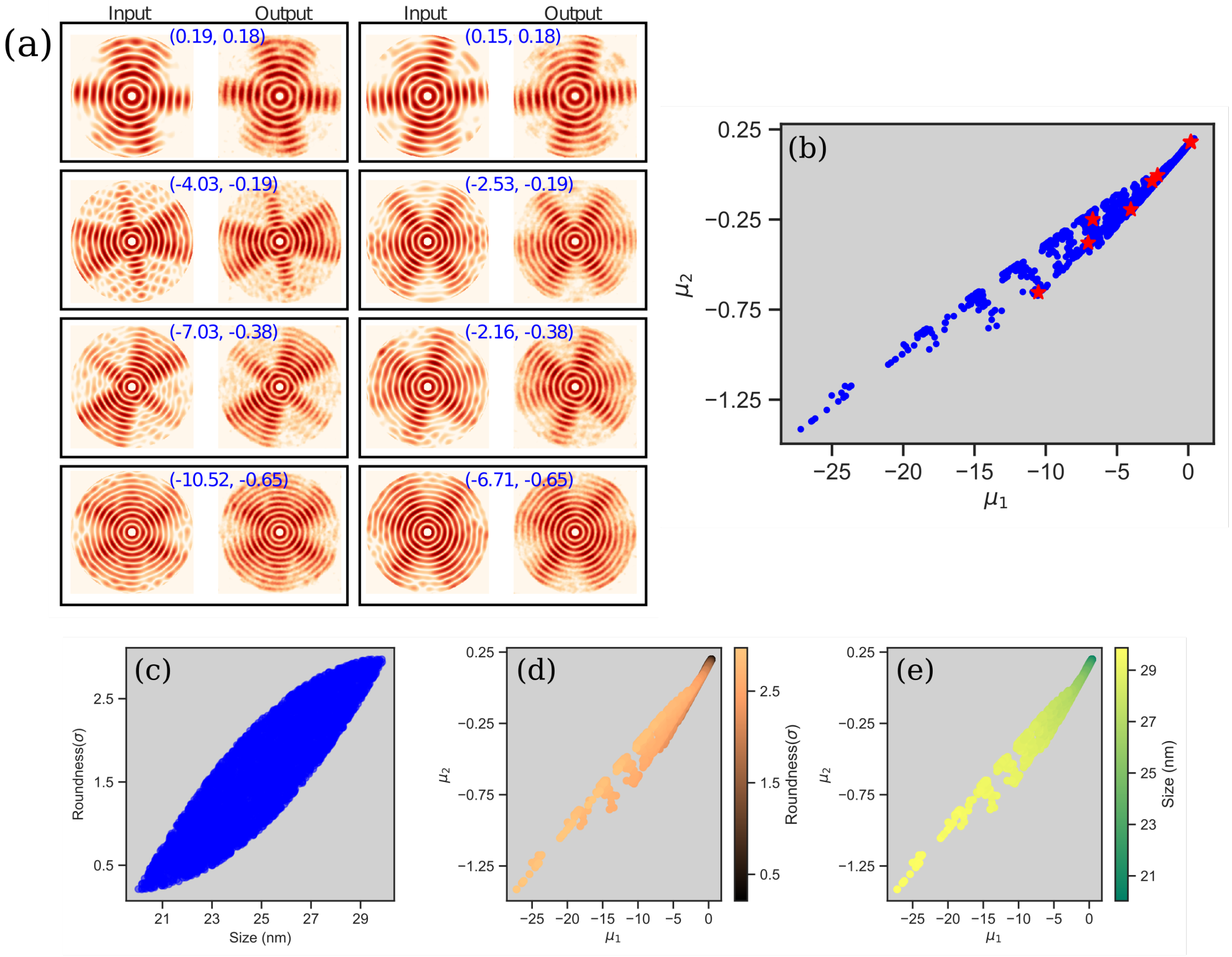

FIG. S9. (a) Comparison between the input 2D intensity data and the corresponding VAE reconstruction on the simulated dataset. (b) Latent space representation of the VAE; the red points indicate the data sample shown in (a), with coordinates provided in the inset. (c) Ground truth distribution of size and roundness parameters used to generate the simulated dataset of icosahedra particles. (d, e) Latent space representations of VAE color-coded by roundness and size, respectively.

4. PERFORMANCE OF VAE ON SIMULATED DATA

In order to test the VAE architecture on a simulated dataset with similar characteristics, 5000 2D intensity images were generated from heterogeneous particles in random orientations. The particles were uniform density icosahedra of variable size and *roundness*, where the latter was implemented by smoothing and thresholding a faceted icosahedron.

The VAE with 2 dimensional latent space was trained for 500 epochs and terminated as the loss reached a minimal, stable value that no longer changed across epochs. Fig. S9(a) illustrates the reconstruction performance of the VAE on simulated 2D intensity data at the final epoch. The majority of features were successfully reconstructed, and the VAE effectively captured the inherent varying features in the input data. Fig. S9(b) displays the latent space representation learned by the VAE on the simulated data. The red points correspond to the reconstructed data sample shown in Fig. S9(a).

Fig. S9(c) presents the distribution of roundness and size parameters used to generate diffraction patterns from the icosahedral particles. This distribution reflects continuous variation in shape and roundness which is controlled by σ. Fig. S9(d) and (e) show the latent space representation learned by the VAE, with data points colored by the ground truth roundness and size, respectively. The latent space effectively captures the relationships between these parameters.

[1] A. Punjani, J. L. Rubinstein, D. J. Fleet, and M. A. Brubaker, cryosparc: algorithms for rapid unsupervised cryo-em structure determination, Nature methods **14**, 290 (2017).

[2] P. B. Rosenthal and R. Henderson, Optimal determination of particle orientation, absolute hand, and contrast loss in single-particle electron cryomicroscopy, Journal of molecular biology **333**, 721 (2003).

[3] K. Ayyer, T.-Y. Lan, V. Elser, and N. D. Loh, Dragonfly: an implementation of the expand–maximize–compress algorithm for single-particle imaging, Journal of applied crystallography **49**, 1320 (2016).

[4] K. Ayyer, P. L. Xavier, J. Bielecki, Z. Shen, B. J. Daurer, A. K. Samanta, S. Awel, R. Bean, A. Barty, M. Bergemann, *et al.*, 3d diffractive imaging of nanoparticle ensembles using an x-ray laser, Optica **8**, 15 (2021).

[5] P. Plevka, K. Tars, and L. Liljas, Crystal packing of a bacteriophage ms2 coat protein mutant corresponds to octahedral particles, Protein Science **17**, 1731 (2008).

[6] P. Plevka, K. Tars, and L. Liljas, Structure and stability of icosahedral particles of a covalent coat protein dimer of bacteriophage ms2, Protein Science **18**, 1653 (2009).

[7] D. S. Peabody and F. Lim, Complementation of rna binding site mutations in ms2 coat protein heterodimers, Nucleic acids research **24**, 2352 (1996).

[8] D. P. Kingma and J. Ba, Adam: A method for stochastic optimization, arXiv preprint arXiv:1412.6980 (2014).
